# Detection of DNA Double Strand Breaks by γH2AX Does Not Result in 53bp1 Recruitment in Mouse Retinal Tissues

**DOI:** 10.3389/fnins.2018.00286

**Published:** 2018-05-01

**Authors:** Brigitte Müller, N. M. Ellinwood, Birgit Lorenz, Knut Stieger

**Affiliations:** ^1^Department of Ophthalmology, Justus-Liebig-University Giessen, Giessen, Germany; ^2^Department of Animal Science and Veterinary Clinical Science, Iowa State University, Ames, IA, United States

**Keywords:** DNA repair, DNA DSB, mouse retina, mouse models, photoreceptor cells, inherited retinopathies, gene editing, immunohistochemistry

## Abstract

Gene editing is an attractive potential treatment of inherited retinopathies. However, it often relies on endogenous DNA repair. Retinal DNA repair is incompletely characterized in humans and animal models. We investigated recruitment of the double stranded break (DSB) repair complex of γH2AX and 53bp1 in both developing and mature mouse neuroretinas. We evaluated the immunofluorescent retinal expression of these proteins during development (P07-P30) in normal and retinal degeneration models, as well as in potassium bromate induced DSB repair in normal adult (3 months) retinal explants. The two murine retinopathy models used had different mutations in *Pde6b*: the severe rd1 and the milder rd10 models. Compared to normal adult retina, we found increased numbers of γH2AX positive foci in all retinal neurons of the developing retina in both model and control retinas, as well as in wild type untreated retinal explant cultures. In contrast, the 53bp1 staining of the retina differed both in amount and character between cell types at all ages and in all model systems. There was strong pan nuclear staining in ganglion, amacrine, and horizontal cells, and cone photoreceptors, which was attenuated. Rod photoreceptors did not stain unequivocally. In all samples, 53bp1 stained foci only rarely occurred. Co-localization of 53bp1 and γH2AX staining was a very rare event (< 1% of γH2AX foci in the ONL and < 3% in the INL), suggesting the potential for alternate DSB sensing and repair proteins in the murine retina. At a minimum, murine retinal DSB repair does not appear to follow canonical pathways, and our findings suggests further investigation is warranted.

## Introduction

Inherited retinal dystrophies are disorders which lead to visual impairment and in severe forms, to blindness, and have an estimated prevalence of 1 in 4,000 (Berger et al., 2010). Because it is immuno-privileged tissue that is easily accessed for therapeutic interventions, the eye is both target for use of gene and cell therapies for inherited disorders, and is also a paradigmatic model system for the development of these approaches for inherited disorders affecting other tissues. While gene replacement therapies can be very efficient for small genes that fit into viral vectors for gene delivery (Bennett, 2017), the advent of genomic editing technologies such as CRISPR-Cas9 has opened new possibilities to target even larger genes at endogenous genomic loci (Maeder and Gersbach, 2016).

The idea of using genome editing to repair disease-causing mutations is comparatively young, and relies on highly specific endonucleases and the capacity of the cell to repair double-strand breaks (DSBs) (Carroll, 2008; Carroll and Beumer, 2014; Gaj et al., 2016; Suzuki et al., 2016). This happens either through the error-prone non-homologous end-joining (NHEJ) pathway or, with high fidelity, through homology-directed repair (HDR) in the presence of a DNA donor template (Jasin and Haber, 2016). An alternative repair pathway called microhomology-mediated end joining (MMEJ) has recently been discovered (Truong et al., 2013).

The predominant DSB repair pathway in mitotic cells at all cell-cycle stages is NHEJ, whereas HDR and MMEJ are only active during the G2 and G1 phases of the cell cycle, respectively (Sakuma et al., 2016). Although robust data exist on the complexity of DNA repair mechanisms in dividing cells *in vitro*, almost nothing is known about post-mitotic neurons such as photoreceptors (PRs). In these cell types the system appears to differ: in mouse rod PRs, DSBs induced by radiation are insufficiently repaired as measured by quantification of repair foci over time (Frohns et al., 2014). This deficit may be associated with changes to the nuclear architecture, as mouse rod PRs exhibit an inverted arrangement of chromatin which is related to the nocturnal nature of rodents and is thought to optimize light perception in the dark (Solovei et al., 2009). In addition, DNA repair activity is also higher in the developing vs. adult mouse retina (Frohns et al., 2014).

Based on *in vitro* data, DNA damage sensing happens through the binding of H2AX to the DSB site and its subsequent phosphorylation (γH2AX), resulting in the recruitment of checkpoint factors to the DSB (Yuan et al., 2010; Georgoulis et al., 2017). The ubiquitinylation of H2A and H2AX then triggers the further binding by early phase DNA repair proteins, amongst which is p53 binding protein 1 (53bp1) which plays an important role in the pathway of repair proceeding by NHEJ. Presence of 53bp1 at the DSB normally results in the recruitment of proteins that are part of the NHEJ pathway and in the inhibition of BRCA1 activity, which is involved in the HDR pathway (Ward et al., 2003; Ginjala et al., 2011). The occurence of γH2AX and 53BP1 at the DSBs results in so-called repair foci (Manis et al., 2004), which can be detected by colocalized antibody binding (Rogakou et al., 1999; Frohns et al., 2014).

The aim of this study is to determine the cell-specific recruitment of γH2AX and 53bp1 in rod and cone photoreceptors in different mouse model systems in order to shed further light on the capacity of the retina to enable genome editing. We also included retinal organ culture in our study as another model for degeneration in the retina. Many alterations observed during *in vitro* retina culturing resemble some characteristics of experimental retinal detachment and diabetic retinopathy *in vivo*, respectively (Fisher et al., 2005; Valdés et al., 2016). We detected regular γH2AX repair foci in wildtype as well as degenerating retinae, but it appears that 53bp1 occurrence at the repair foci is not the normal downstream process, indicating cell specific characteristics in these highly-specialized cells.

## Materials and methods

### Experimental design

Overall, 15 adult animals and 16 immature mice were included in this study comprising both females and males. At least three eyes from different individuals were used for histologic analysis of each explant culture period. Immature rd1 and C3H mice were investigated at ages post-natal day (p) 07, p13, p21, p28. Immature rd10 and C57BL/6J were investigated at p14, p18, p24 and p30. At least two immunostaining procedures were performed for each antibody and each investigated mouse tissue of the respective mouse lines and ages. For microscopic analysis, 2–3 sections of each immunostaining per respective mouse line and age were analyzed.

### Animal handling and ethics statement

Three and nine months old wild type C57BL/6J mice (Jackson stock # 000664, Charles River, Germany) were used in this study. In addition, immature rd1 mice (RRID:MGI:3803328), rd10 mice (RRID:MGI:3581193) and their respective wildtype strains C3H and C57BL/6J were subjects in this study and were provided by François Paquet-Durand from Tübingen. Animals were housed and bred in the animal facility of the University of Giessen under standard white cyclic lighting, had free access to food and water. Day of birth was considered as post-natal day 0. All procedures concerning animal handling and sacrificing complied with the European legislation of Health Principles of Laboratory Animal Care in accordance with the ARVO statement for the Use of Animals in Ophthalmic and Vision Research. The protocol was approved by the Institutional Animal Care and Use Committee (IACUC) of the Justus-Liebig-University (TV-No M_474). All efforts were made to minimize the number of animals used and pain and distress.

### Preparation of organotypic retina culture

Three and nine month old C57BL/6J retinae were used to generate organotypic retina culture as described previously (Müller et al., 2017). In brief, explanted retinae were cultured on track etched polycarbonate membrane (TEPC), pore size 0.4 μm and 30 mm in diameter (#35006, Laboglob.com GmbH, Germany) with the photoreceptor layer facing the supporting membrane. Inserts were put into six-well culture plates and incubated in complete medium with supplements at 37°C. Every second day the full volume of complete medium, 1.2 ml per well, was replaced with fresh medium. The culture period was ended by immediate fixation in 4% paraformaldehyde in phosphate buffered saline (PBS) for 45 min at time points ranging from 2 to 10 days. Fresh, i.e., un-cultured retinas were used as controls.

### KBrO_3_ incubation procedure (positive control for DNA DSBs)

To initiate DNA DSBs, whole adult C57BL/6J mouse retina was dissected in Hanks' Balanced Saline Solution (GIBCO® HBSS; #14025076, Thermo Fisher, Germany), transferred into a small Petri dish (30 mm in diameter), and incubated in 1.5 mM KBrO_3_ (#4396, Roth, Germany) for 1.5 h at 37°C. Organotypic retina cultures of 2, 4, 6, and 8 days were treated likewise. Working solution of KBrO_3_ was prepared with pure water. Respective control retinae were treated with plain water only. Subsequently, all treated retinal tissues were briefly rinsed in HBSS and immediately fixed in 4% paraformaldehyde in PBS at room temperature for 45 min. After washing in PBS, all treated retinal tissue was cryo-protected in graded sucrose solutions (10, 20, and 30% in PBS), frozen, cut, and immunostained with γH2AX antibodies (see next section).

### Tissue processing and immunohistochemistry

For frozen sectioning, immature and adult mouse eyecups and retinal explants were treated as described previously (Müller et al., 2017). Immunostaining was performed employing the two-step indirect method. Prior to 53bp1 antibody staining, antigen retrieval was performed (Eberhart et al., 2012). Sections were incubated at room temperature overnight in primary antibodies (see Table 1). Immunofluorescence was performed using Alexa Fluor 488-conjugated secondary antibodies (#21202, Thermo Fisher Scientific, Germany) or Alexa 594 (#21207, Thermo Fisher Scientific, Germany).

**Table 1:** Primary antibodies.

**Antigen**	**Description of immunogen**	**Source, host species, Cat. #, clone or lot #, RRID #**	**Concentration used**
γH2AX	Peptide (C-KATQA[pS]QEY) corresponding to amino acids 134-142 of human histone H2A.X	Millipore, mouse monoclonal, Cat# 05-636, Clone JBW301, RRID:AB_309864	1:500
γH2AX	Synthetic phosphopeptide corresponding to residues surrounding Ser139 of human H2A.X.	Cell Signaling Technology, rabbit monoclonal, Cat# 9718, Clone 20E3, RRID:AB_2118009	1:3,000
53BP1	Between residue 350 and 400 of human tumor protein p53 binding protein 1, NP_005648.1 (GeneID 7158).	Bethyl, rabbit polyclonal, Cat# IHC-00053, RRID:AB_2206769	1:300
LaminB_2_	66 kD lamin B2 isoform of LaminB_2_	Thermo Fisher Scientific, mouse monoclonal,Cat# 33-2100, Clone: E-3 RRID:AB_2533107	1:200
Calbindin	Recombinant rat calbindin D-28k	Swant, rabbit polyclonal, Cat# CB-38a, Lot 9.03	1:1,000–1:100,000

### Laser scanning confocal microscopy

Confocal images were taken using an Olympus FV10i confocal microscope, equipped with Argon and HeNe lasers. High-resolution scanning of image stacks was performed with an UPlanSApo x60/1.35 (Olympus) oil immersion objective at 1,024 × 1,024 pixels and a z-axis increment of 0.3 μm. For analysis of immunolabeled cells and their processes, a stack of 2–12 sections was taken (0.7-μm z-axis step size). Cell processes were reconstructed by collapsing the stacks into a single plane. Brightness and contrast of the final images were adjusted using Adobe Photoshop CS5 (San Jose, CA).

### Quantification of γH2AX and 53bp1 immunoreactive foci

Quantification of γH2AX and 53bp1 immunoreactive foci was performed on vertical frozen sections of developing retina of rd1, rd10 mice and their respective wildtype C3H and C57BL/6J. An area of 2320 μm^2^ was defined in each image in the outer nuclear layer (ONL) and in the inner nuclear layer (INL), and all γH2AX and 53bp1 immunoreactive foci within that square were counted. Only central retinal regions were analyzed. Foci co-localizing 53bp1 and γH2AX immunoreactivity were given as percentage of all γH2AX immunoreactive foci in the respective field. At least three micrographs per time point and mouse line were analyzed. Image stacks 2 μm in depth were taken.

### Statistical analysis

Statistical comparisons among different experimental groups were made using a two-tailed Student's *t*-test and SigmaPlot 12 software. Error bars indicate SD.

## Results

### Localization of γH2AX within the murine wildtype retina

We analyzed retinal tissue from two wild type mouse lines, C3H and C57BL/6J, because the subsequently analyzed models with hereditary retinal degeneration are based on the two lines (rd1 is based on C3H, rd10 is based on C57BL/6J).

**Figure 1:**
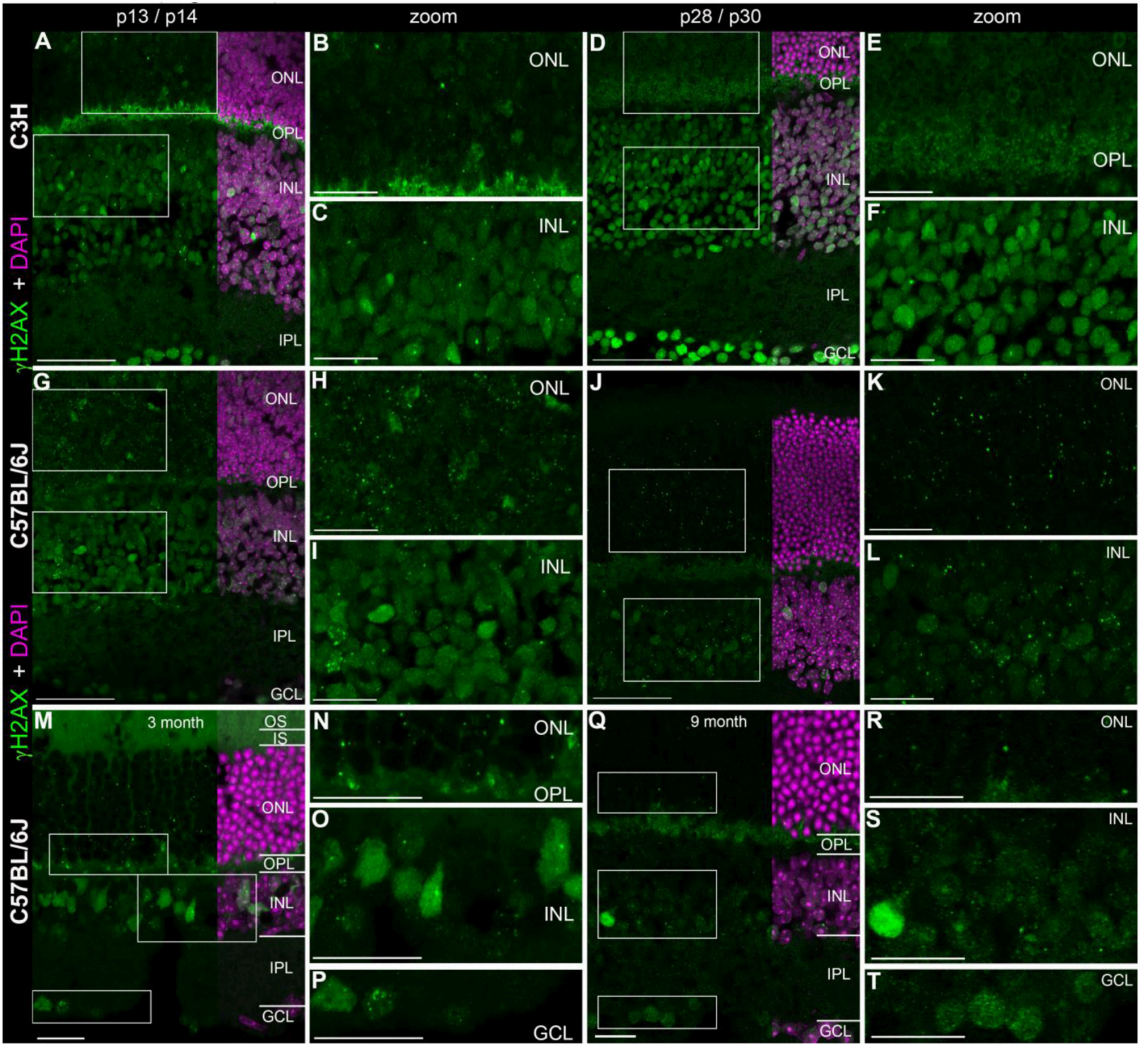
Presence of γH2AX in wildtype retina at different ages. **(A)** Entire retina and magnifications of ONL **(B)** and INL **(C)** of C3H mice at 2 weeks of age. **(D)** Entire retina and magnifications of ONL **(E)** and INL **(F)** of C3H mice at 4 weeks of age. **(G)** Entire retina and magnifications of ONL **(H)** and INL **(I)** of C57BL/6J mice at 2 weeks of age. **(J)** Entire retina and magnifications of ONL **(K)** and INL **(L)** of C57BL/6J mice at 4 weeks of age. **(M)** Entire retina and magnifications of ONL **(N)**, INL **(O)**, and GCL **(P)** of C57BL/6J mice at 3 months of age. **(Q)** Entire retina and magnifications of ONL **(R)**, INL **(S)**, and GCL **(T)** of C57BL/6J mice at 9 months of age. For detailed description see Results. IS, inner segments; ONL, outer nuclear layer; OPL, outer plexiform layer; INL, inner nuclear layer; IPL, inner plexiform layer; GCL, ganglion cell layer. Scales **A,D,G,J**: 50 μm, scales **B,C,E,F,H,I,K,L,M–T**: 20 μm.

In the developing mouse retina, defined γH2AX immunoreactive foci were found numerously in the inner (INL) and outer nuclear layers (ONL) (Figures 1A–L). The occurrence of several foci per nucleus seemed quite common especially in the C57BL/6J mice. At p14, pan nuclear staining of γH2AX was visible in some nuclei of the INL and ONL (Figures 1B,C,H,I). In the mature mouse retina at 3 and 9 month of age, γH2AX immunoreactive foci were found in all nuclear layers but appeared to be less frequent than in the developing retina (Figures 1G–T). Individual nuclei showed γH2AX immunoreactive pan nuclear staining in the INL and GCL. In the ONL, no pan nuclear staining was found in the mature mouse retina. In the OPL of C3H and adult C57BL/6J mice, γH2AX immunoreactivity was observed with the polyclonal antinserum against γH2AX (Figures 1A,B,D,E,Q). It was viewed as unspecific background staining related to the polyclonal γH2AX antiserum (see Table 1). Specimen treated with the monoclonal γH2AX antibody did not show γH2AX-immunoreactivity in the OPL.

**Figure 2:**
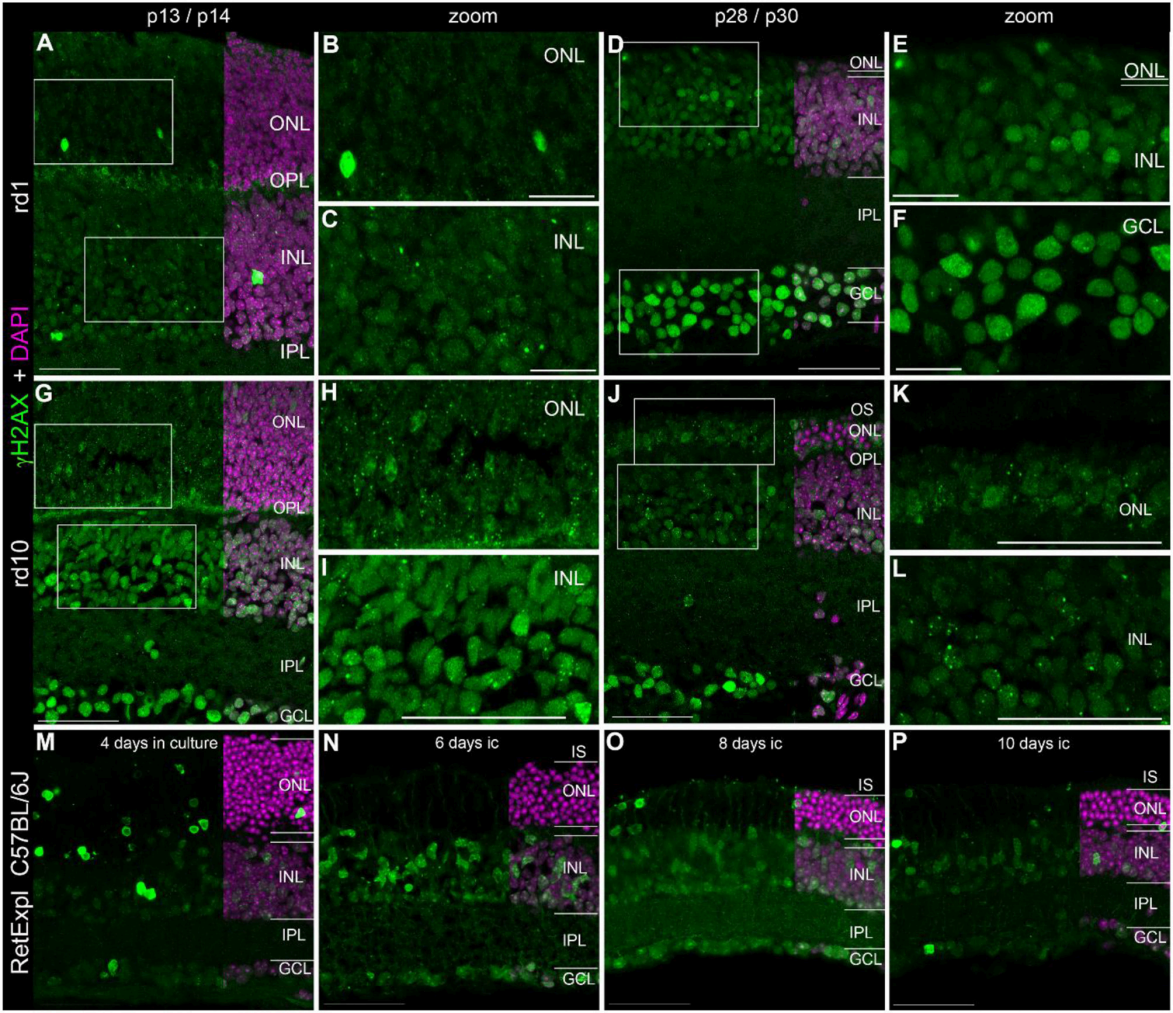
Presence of γH2AX in the degenerating retina. **(A)** Entire retina and magnifications of ONL **(B)** and INL **(C)** of rd1 mice at 2 weeks of age. **(D)** Entire retina and magnifications of ONL+INL **(E)** and GCL **(F)** of rd1 mice at 4 weeks of age. **(G)** Entire retina and magnifications of ONL **(H)** and INL **(I)** of rd10 mice at 2 weeks of age. **(J)** Entire retina and magnifications of ONL **(K)** and INL **(L)** of rd10 mice at 4 weeks of age. **(M–P)** Entire retina of 3 months old C57BL/6J mice taken into culture for 4 days **(M)**, 6 days **(N)**, 8 days **(O)**, and 10 days **(P)**. For detailed description see Results. IS, inner segments; ONL, outer nuclear layer; OPL, outer plexiform layer; INL, inner nuclear layer; IPL, inner plexiform layer; GCL, ganglion cell layer. Scales **A,D,G–P**: 50 μm, scales **B,C,E,F**: 20 μm.

Quantification of γH2AX immunoreactive foci in the ONL and INL of immature (p13/p14) and mature (p28/p30) C3H, C57BL/6J, rd1, and rd10 mouse retina revealed highest foci numbers in the ONL (Data not shown). Except for the C3H mouse retina, immature and mature retina showed no significant difference in the number of γH2AX immunoreactive foci in the INL. In the ONL, differences in the number of γH2AX immunoreactive foci were not significant at immature or mature ages. At 4 weeks of age, quantification of γH2AX immunoreactive foci was not possible due to loss of most photoreceptors in the ONL in the rd mouse lines (Figures 2D,J).

### Localization of γH2AX in degenerating retina

In addition to the rd1 and rd10 mouse lines, we also analyzed tissue from organotypic retina culture (C57BL/6J) as an alternative model of retinal degeneration.

In the developing retina of rd1 and rd10 mice, γH2AX immunoreactive foci were found in the INL and ONL (Figures 2A–L). The occurrence of several foci per nucleus seemed quite common especially in the rd10 mice. Similar to wt retinae at p14, pan nuclear staining of γH2AX was visible in some nuclei of the INL and ONL (Figures 2A–C,G–I). At p30, when PR degeneration has progressed a lot in both mouse lines, many cells of the GCL showed pan nuclear staining of γH2AX (Figures 2D,J). In the organotypic retina culture, pan nuclear staining was even more prominent than defined γH2AX immunoreactive foci (Figures 2M–P). After 4 days of culture, pan nuclear staining was found in all nuclear layers but appeared to be most frequent in the ONL (Figure 2M). In the GCL, only individual nuclei showed pan nuclear staining of γH2AX. In the retinae of 2 week old rd1 and rd10 mice, γH2AX-immunoreactive background staining was observed in the OPL (Figures 2A,G,H).

### Localization of P53 binding protein 1 (53bp1) within the murine wildtype retina

**Figure 3:**
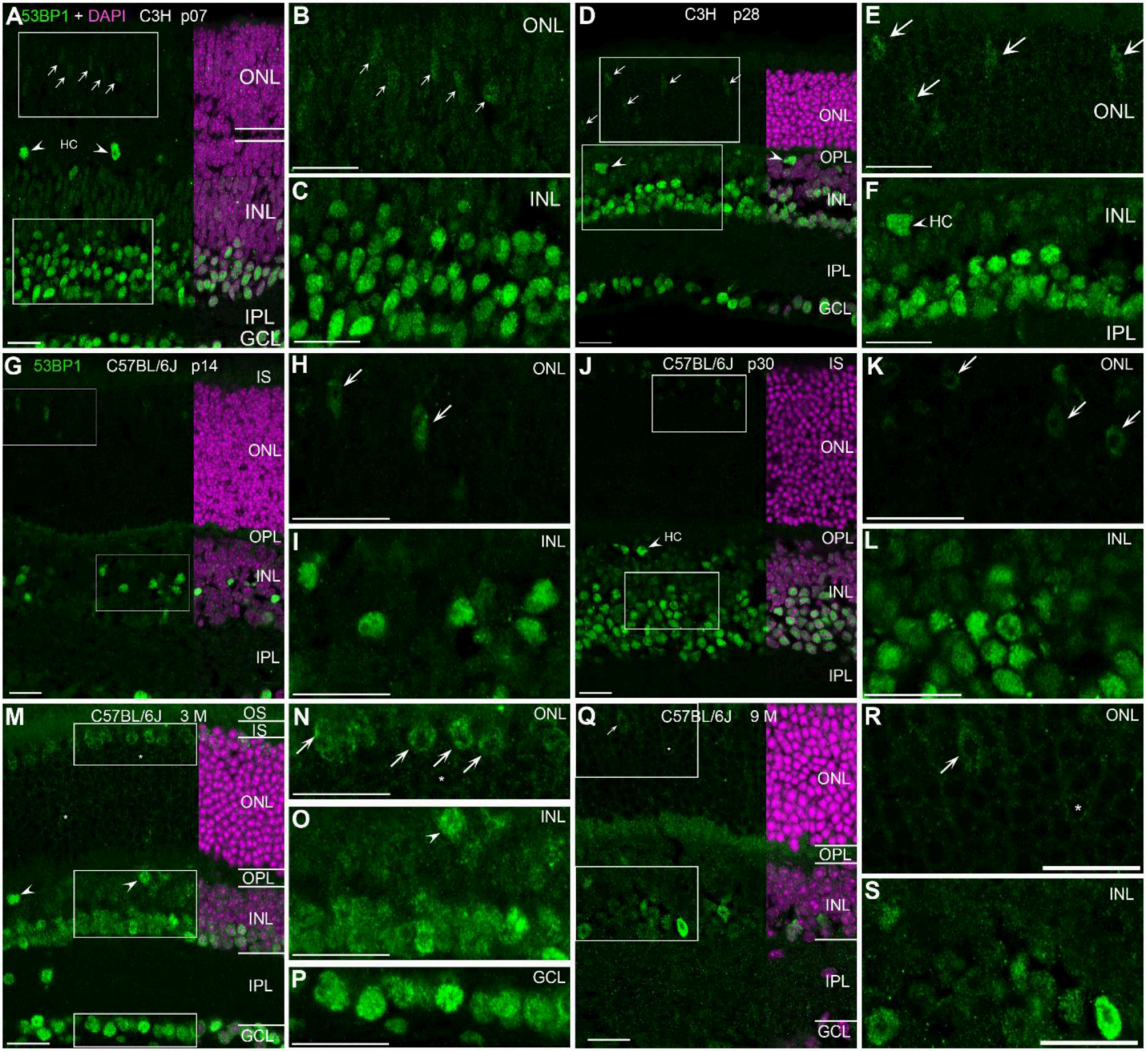
Presence of 53bp1 in wildtype retina at different ages. **(A)** Entire retina and magnifications of ONL **(B)** and INL **(C)** of C3H mice at 1 week of age. Arrows in **(A)** indicate cone photoreceptor nuclei. **(D)** Entire retina and magnifications of ONL **(E)** and INL **(F)** of C3H mice at 4 weeks of age. Arrows in **(D,E)** indicate cone photoreceptor nuclei. Arrow heads in **(D,F)** indicate horizontal cell nuclei (HC). **(G)** Entire retina and magnifications of ONL **(H)** and INL **(I)** of C57BL/6J mice at 2 weeks of age. Arrows in **(H)** indicate cone photoreceptor nuclei. **(J)** Entire retina and magnifications of ONL **(K)** and INL **(L)** of C57BL/6J mice at 4 weeks of age. Arrows in **(K)** indicate cone photoreceptor nuclei. **(M)** Entire retina and magnifications of ONL **(N)**, INL **(O)**, and GCL **(P)** of C57BL/6J mice at 3 months of age. Arrows in **(N)** indicate cone photoreceptor nuclei. **(Q)** Entire retina and magnifications of ONL **(R)**, and INL **(S)** of C57BL/6J mice at 9 months of age. Arrows in **(Q,R)** indicate cone photoreceptor nuclei. For more detailed description see Results. HC, horizontal cell; IS, inner segments; ONL, outer nuclear layer; OPL, outer plexiform layer; INL, inner nuclear layer; IPL, inner plexiform layer; GCL, ganglion cell layer. All Scales 20 μm.

With the exception of the developing retina at p07, many nuclei of the INL and GCL showed generally high level of immunoreactivity to 53bp1 (Figures 3A–C). At p14 in C57BL/6J, nuclei with strong labeling were present only in the inner retina (Figures 3G–I). In the retina of p30 mice and adult ages, the intensities of the immunostaining varied between cell types in the different retinal layers (Figures 3D–F,J–S). In the GCL, most nuclei showed bright immunoreactivity (Figures 3M,P). Interestingly, the brightly labeled nuclei in the INL were restricted to the inner third, irrespective of age (Figures 3D,J,M). Most of these nuclei belong to amacrine cells, some may be Müller cells. Individual large nuclei close to the OPL showed bright immunoreactivity too (arrow heads in Figures 3A,D,F,J,M,O). Due to their size, location and low occurrence, i.e., big intervals between individual nuclei, staining is consistent with horizontal cells. Double immunostainings with 53bp1 and pATM antibodies clearly revealed the horizontal cell morphology (data not shown). The remaining nuclei in the outer half of the INL showed moderate to light immunoreactivity and are consistent with bipolar cells (Figures 3A,D,J,M). With ongoing maturation, occurrence of bright immunofluorescence was clearly localized to the inner two rows of INL nuclei and all GCL nuclei. In the ONL, cone nuclei showed comparably bright immunolabeling (arrows in Figures 3E,H,K,M). Double immunolabeling of 53bp1 and calbindin antibodies confirmed 53bp1 localization in amacrine and horizontal cells in all investigated mouse lines (Supplemental Figure 3). As well, co-localization of glutamine synthetase in Müller glia cells was confirmed by double immunolabeling of 53bp1 and glutamine synthetase (data not shown). Immunoreactive cone nuclei were found at all ages investigated. Due to the inverted chromatin distribution in rods, 53bp1 immunoreactivity was localized very close to the nuclear envelope forming a lightly stained thin ring around the heterochromatin (examples are marked by asterisk in Figures 3M,N,Q,R). The heterochromatin in rods was brightly stained by DAPI. Nine-month-old mouse retina revealed less intense immunoreactivity in the inner retina, i.e., INL and GCL (Figures 3Q,S).

### Localization of 53bp1 in degenerating retina

**Figure 4:**
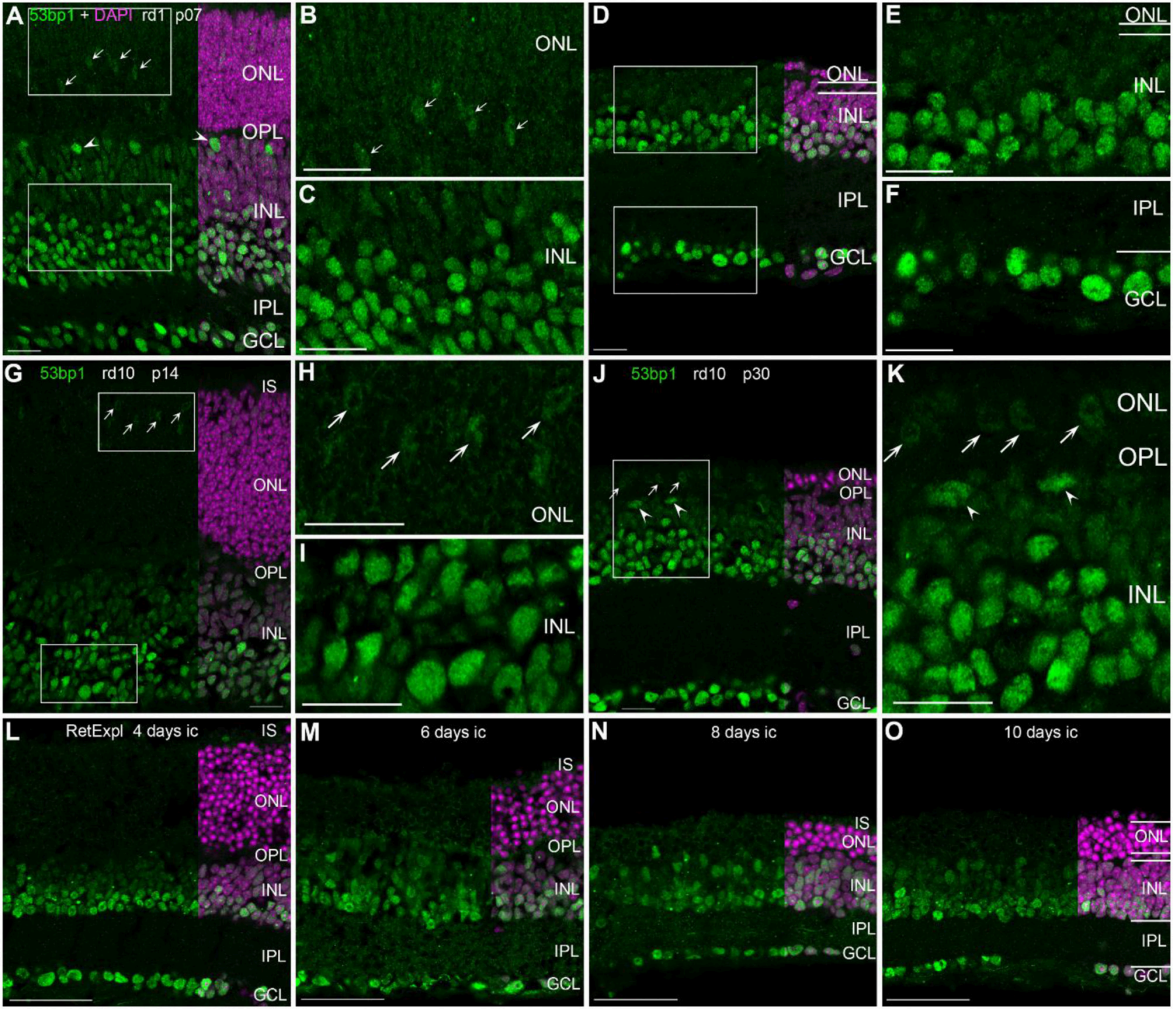
Presence of 53bp1 in the degenerating retina. **(A)** Entire retina and magnifications of ONL **(B)** and INL **(C)** of rd1 mice at 1 week of age. Arrows in **(A)** indicate cone photoreceptor nuclei. Arrow heads in **(A)** indicate nuclei of horizontal cells. **(D)** Entire retina and magnifications of ONL+INL **(E)** and GCL **(F)** of rd1 mice at 4 weeks of age. **(G)** Entire retina and magnifications of ONL **(H)** and INL **(I)** of rd10 mice at 2 weeks of age. **(J)** Entire retina and magnification of ONL+INL **(K)** of rd10 mice at 4 weeks of age. **(L–O)** Entire retina of 3 months old C57BL/6J mice taken into culture for 4 days **(L)**, 6 days **(M)**, 8 days **(N)**, and 10 days **(O)**. For detailed description see Results. IS, inner segments, ONL, outer nuclear layer; OPL, outer plexiform layer; INL, inner nuclear layer; IPL, inner plexiform layer; GCL, ganglion cell layer. Scales **A–K**: 20 μm, scales **L–O**: 50 μm.

In the developing retina of rd1 and rd10 mice, 53bp1 immunoreactivity was bright and localized in many nuclei of the INL and GCL (Figures 4A–K). At early ages p07 and p14, nuclei of the same cell types were as brightly labeled as those in the respective wildtype mice, i.e., amacrine cells, ganglion cells, horizontal cells, and cones (Figures 4A,G). With maturation and progress in degeneration of the outer retina, bright immunolabeling remained to be localized to the inner retina as in the wildtype mice (Figures 4D–F,J,K). At p28 in rd1 mice, degeneration of the ONL was quite advanced and the remaining cones showed very faint immunofluorescence (Figure 4E). In contrast, cones were clearly present and positively labeled in the rd10 mouse retina at p30 (Figure 4K, arrows). In the organotypic retina culture of 3 month-old mice, immunoreactivity was brightly localized to many nuclei of the INL and GCL (Figures 4L–O). Up to 4 days in culture, the occurrence of bright labeling was clearly restricted to nuclei of the inner INL and all GCL nuclei (Figure 4L). With increasing time in culture, intense immunoreactivity dispersed through the whole INL, i.e., the labeling of bipolar cell nuclei in the inner half of the INL was increased after 6 days in culture (Figures 4M–O). Furthermore, moderately stained thin rings around the heterochromatin of rod photoreceptors was observed (Figures 4M–O).

**Figure 5:**
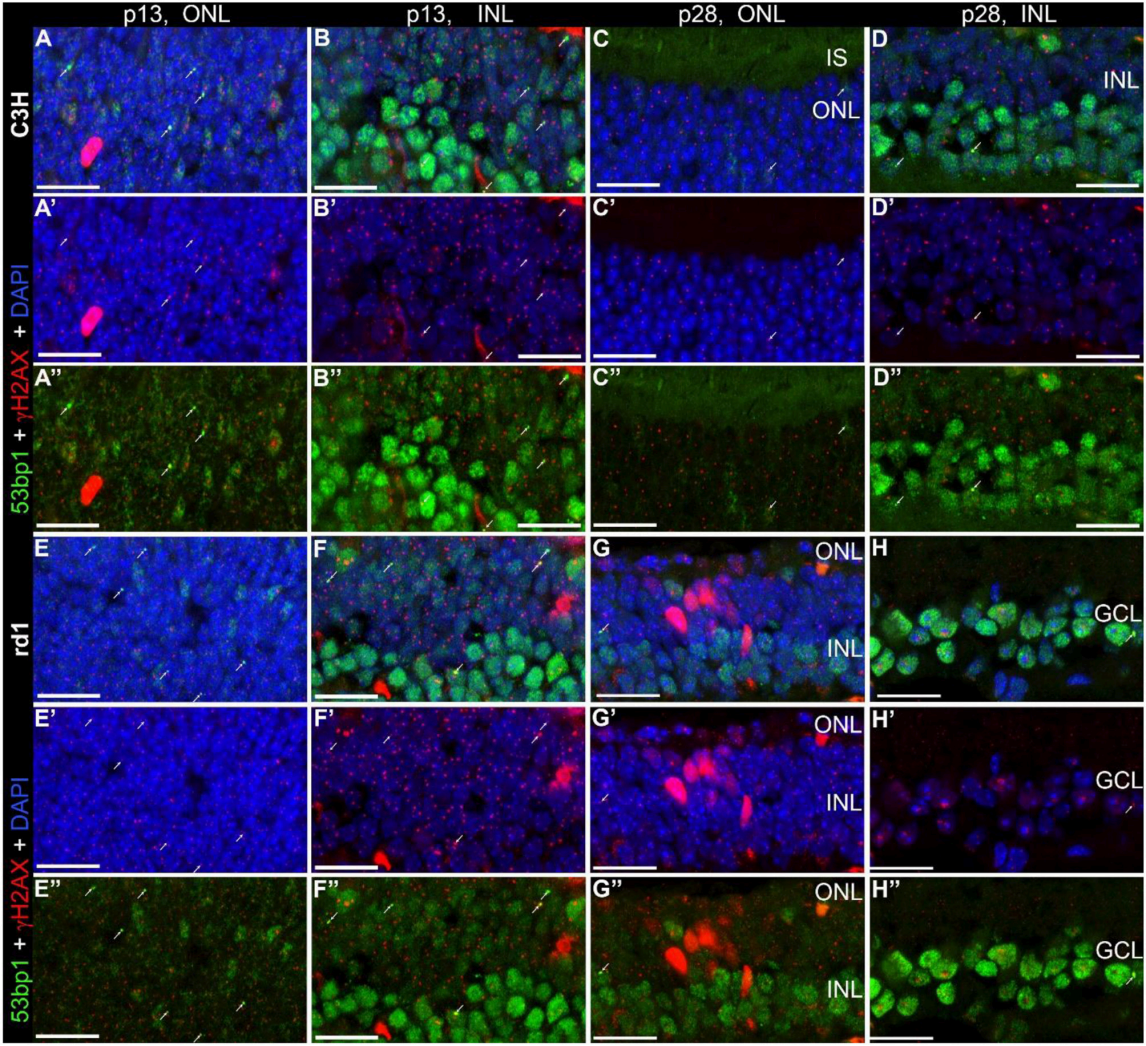
Co-localization of γH2AX and 53bp1 in retinal tissue of C3H and rd1 mice. Vertical frozen sections of developing retina of C3H and rd1 mice were stained with a cocktail of anti γH2AX and 53bp1 antibodies. Only INL and ONL are shown at high magnification. **(A–D”)** C3H mice at p13 and p28. **(E–H”)** rd1 mouse retina at p13 and p28. Numerous γH2AX immunoreactive foci are visible throughout the nuclear layers. 4–6 double labeled foci per field are visible at p13 in both mouse lines (arrows). At p28 only one or two double labeled foci per field are detectable (arrows). Co-localization of γH2AX immunoreactive foci and 53bp1 pan nuclear staining in the INL of C3H and rd1 mice at p13 are presented in Figure 5-1 of extended data. ONL, outer nuclear layer; INL, inner nuclear layer; GCL, ganglion cell layer. All scales = 20 μm.

Following the characterization of the localization of the two proteins separately, we investigated whether γH2AX and 53bp1 are co-localized to the same DNA damage site. Double immunostaining on the developing retina of C3H and rd1 mice showed only very few double labeled foci in the INL and ONL (arrows in Figures 5A–H”). At the same time, numerous γH2AX immunoreactive foci were visible throughout the nuclear layers in C3H at p13 and p28, and rd1 mice at p13, resulting in co-localization of γH2AX positive foci in nuclei with pan nuclear 53bp1 staining in the INL (Figures 5B”,D”,F”; Supplemental Figure 1).

**Figure 6:**
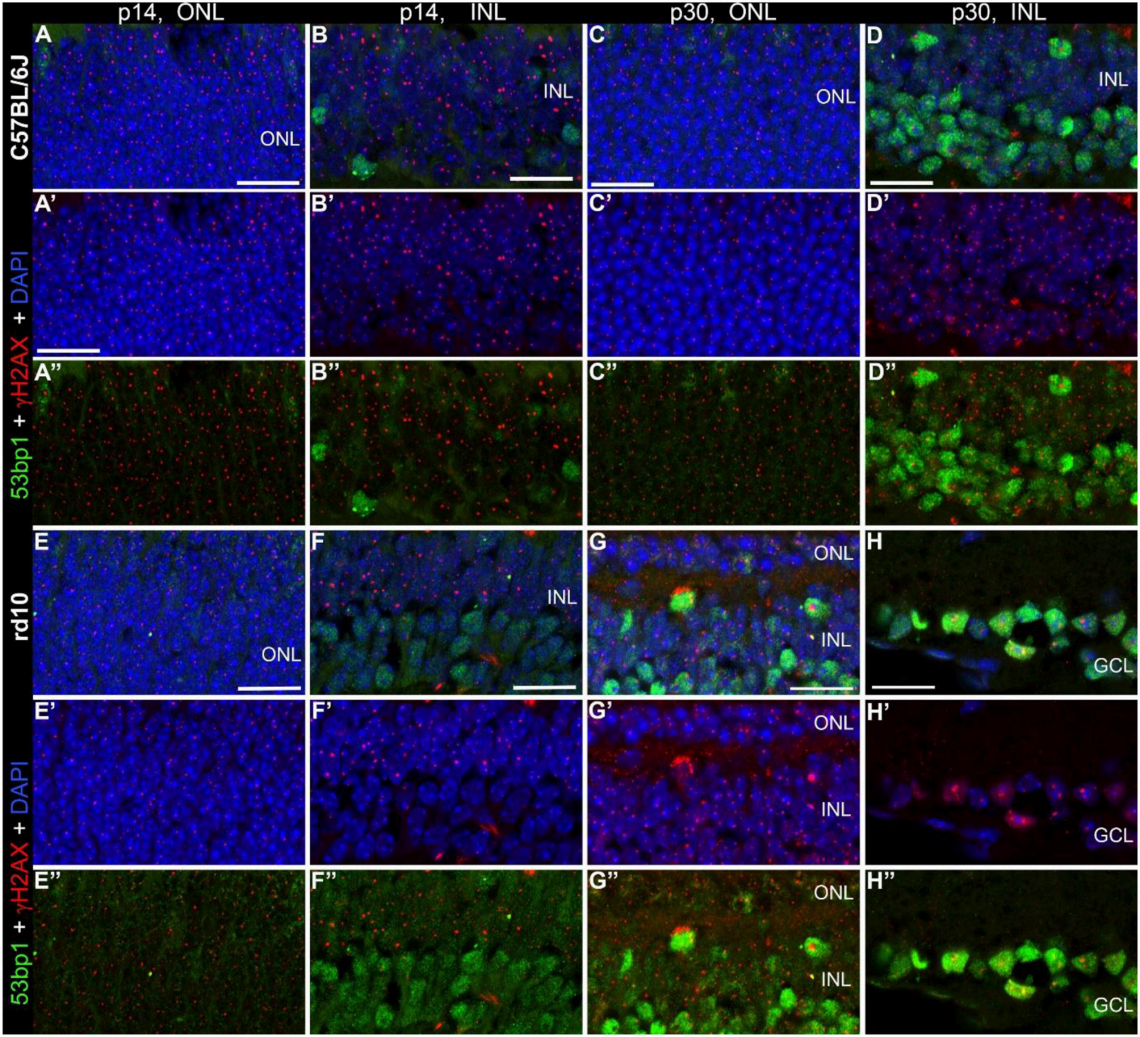
Co-localization of γH2AX and 53BP1 in retinal tissue of C57Bkl6/J and rd10 mice. Vertical frozen sections of developing retina of C57BL/6J and rd10 mice were stained with a cocktail of anti γH2AX and 53BP1 antibodies. Only INL and ONL are shown at high magnification. **(A–D”)** C57BL/6J mice at p14 and p30. **(E–H”)** rd10 moue retina at p14 and p30. Numerous γH2AX immunoreactive foci are visible throughout the nuclear layers. 2–5 double labeled foci per field are visible at both ages, p14 and p30 (arrows). In the INL slightly fewer double labeled foci per field are detectable. ONL, outer nuclear layer; INL, inner nuclear layer; GCL, ganglion cell layer. All scales = 20 μm.

**Figure 7:**
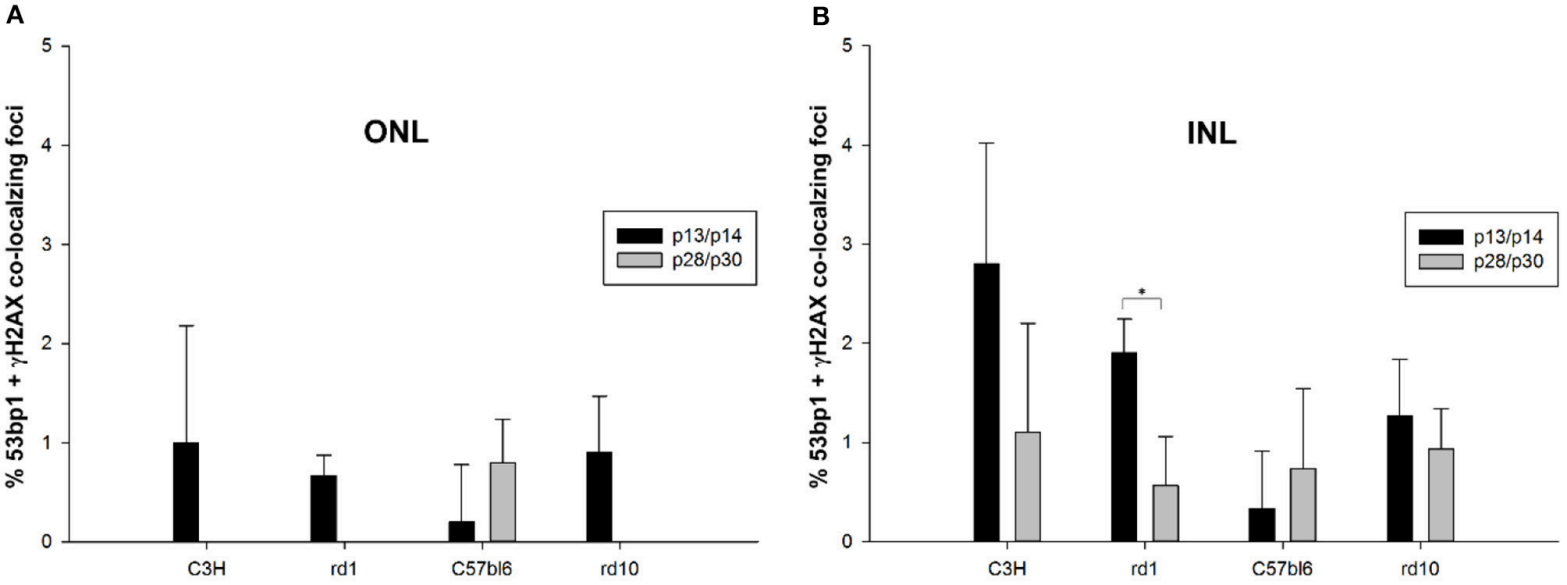
Quantification of immunoreactive foci co-localizing γH2AX and 53bp1 in the ONL and INL of immature (p13/p14) and mature (p28/p30) rd1, rd10, C3H, and C57BL/6J mouse retina. Foci co-localizing 53bp1 and γH2AX immunoreactivity were given as percentage of all γH2AX immunoreactive foci in the respective area. **(A)** In the ONL, occurrence of co-localizing γH2AX and 53bp1 foci was a very rare event (<1% of γH2AX foci) and was only found in the immature age group. At 4 weeks of age, only C57BL/6J mouse retina showed individual co-localizing foci. Due to degeneration of photoreceptors in the rd mouse lines, no co-localizing foci could be found in the ONL. **(B)** In the INL, occurrence of co-localizing γH2AX and 53bp1 foci was 2-fold higher than in the ONL, but still a very rare event (<3% of γH2AX foci). It was significantly higher in the immature age group of rd1 mice. Immature C3H mouse retina showed the highest and C57BL/6J mouse retina the lowest occurrence of γH2AX and 53bp1 foci co-localization in the INL. ^*^p < 0.05.

Double immunostaining in the developing retina of C57BL/6J and rd10 mice showed similar results compared to retinae of C3H and rd1 mice, i.e., very few double labeled foci in the INL and ONL (arrows in Figures 6A–H”). Comparison of the two mouse lines and their respective models of degeneration gave a general impression of more co-localized foci in the C3H and rd1 mouse line (Figure 5). Quantification of the few double labeled foci in the INL and ONL confirmed the impression gained through qualitative analysis of the tissue (Figure 7). The comparison between immature (p13/p14) and mature (p28/p30) mouse retina showed double labeled foci mainly in the immature age group (Figures 7A,B). In the ONL, occurrence of double labeled foci was a very rare event (<1% of γH2AX foci) and found only in the immature age group (Figure 7A). In the retina of the rd mouse lines, no co-localizing foci could be found in the ONL. In the INL of immature rd1 mice, co-localization was significantly higher than in mature rd1 mouse retina (Figure 7B). In immature retina of the wildtype mouse lines, the C3H mouse retina showed the highest and C57BL/6J mouse retina the lowest occurrence of double labeled foci in the INL. However, in the INL, occurrence of co-localizing γH2AX and 53bp1 foci was 2-fold higher than in the ONL, but still a very rare event (< 3% of γH2AX foci).

In summary, our results showed that generally, 53bp1 is not recruited to DSB repair foci positive for phosphorylated H2AX in the ONL and INL, but only occasionally co-localizes. In the INL and GCL, many γH2AX positive foci seem to be localized to the same nucleus as 53bp1, the latter as pan nuclear staining.

### Co-localization of γH2AX and 53bp1 to induced DSB

**Figure 8:**
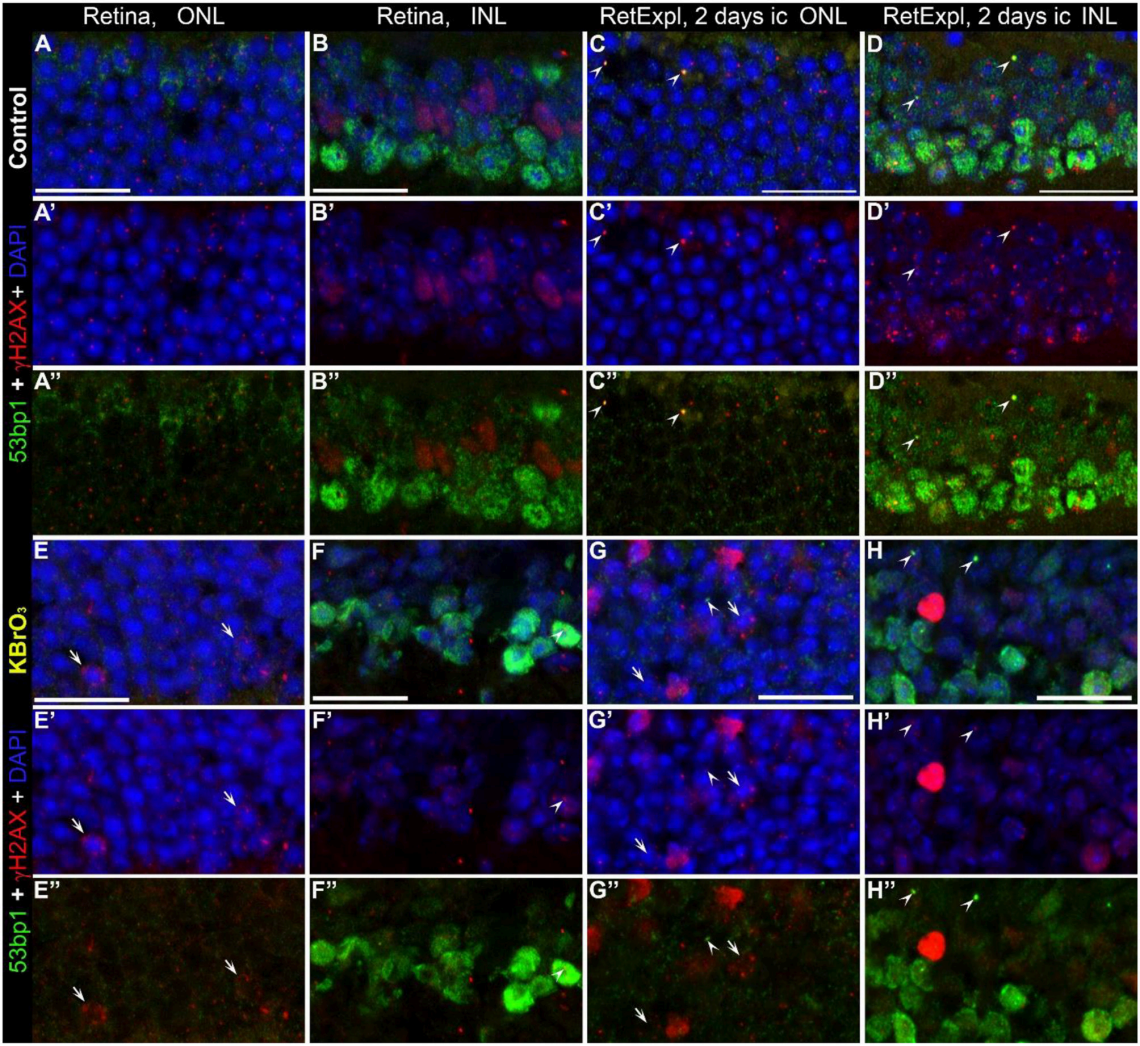
Co-localization of γH2AX and 53bp1 following double strand break induction with KBrO3. γH2AX and 53BP1 double labeled foci were found only in retinal explant culture, in both the KBrO_3_ treated and untreated preparations (the two right pannels, arrow heads). Nuclei with several γH2AX immunoreactive foci were found in the ONL of KBrO3 treated tissue only (arrows). DAPI counterstaining (blue) reveals the chromatin in relation to the location of γH2AX and 53BP1 immunoreactive foci in the nuclei. **(A–B”)** Vertical frozen sections of control retina of 3-month-old mice. **(E–F”)** Sections of 3-month-old mice after KBrO_3_ incubation. **(C–D”)** Sections of control retina of retinal explants after 2 days in culture. **(G–H”)** Sections of retinal explants treated with KBrO_3_ after 2 days in culture. ONL, outer nuclear layer; INL, inner nuclear layer. All scales = 20 μm.

Potassium bromate (KBrO_3_) is an oxidizing agent used as a food additive, which causes kidney damage as a potent nephrotoxic agent, and the mechanism is explained by the generation of oxygen free radicals that induce many DSB and thus cause genomic instability leading to apoptosis (Bao et al., 2008). Here we incubated whole wildtype mouse retina (Figures 8E,F) as well as tissue from organotypic retina culture (Figures 8G,H) in 1.5 mM KBrO_3_ to initiate DSBs. Control retinae were treated with plain water only (Figures 8A–D). While the number of γH2AX foci increased in all samples treated with potassium bromate, 53bp1 immunoreactive foci were only observed in retinal explant culture, in both the KBrO_3_ treated and untreated preparations as single events. Hence, double labeled foci could be found only in retinal explant culture (arrow heads in Figures 8C,D,G,H). Double labeled foci in the ONL and INL did not increase due to the potassium bromate treatment. Nuclei with several γH2AX immunoreactive foci were only found in the ONL of KBrO_3_ treated tissue (arrows in Figures 8E,G). Moderate pan nuclear γH2AX staining was visible in the ONL and bright staining in some nuclei of the INL of treated retinal explant culture (Figures 8G,H). In the INL of the control retinal tissue, a population of nuclei showed moderate pan nuclear γH2AX staining (Figures 8B,B”). Pan nuclear immunostaining of 53bp1 was not altered due to KBrO_3_ treatment in the nuclear layers.

In summary, KBrO_3_ treatment only increased the number of γH2AX foci but had no effect on the number of immunoreactive foci or the pan nuclear staining of 53bp1.

### Intranuclear localization of 53bp1

We used lamin B_2_ antiserum to label the lamina of the inner membrane of the nuclear envelope. The nuclear lamina consists of a layer of four distinct lamin proteins which are in close apposition to the nucleoplasmic surface of the inner nuclear membrane (Aebi et al., 1986).

**Figure 9:**
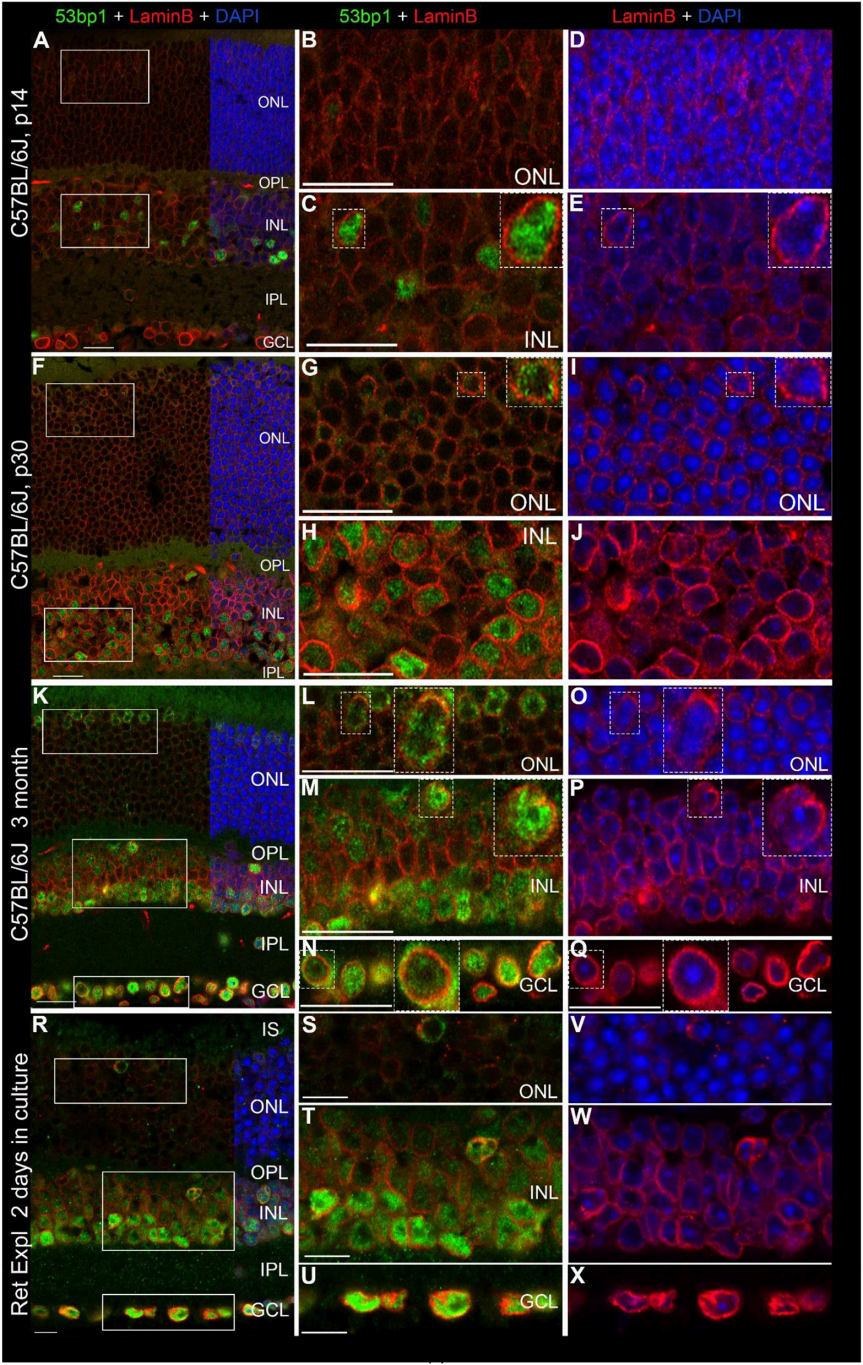
Localization of 53bp1 immunoreactivity with regard to the nuclear membrane. Double immunostaining with anti 53bp1 (green) and lamin B_2_ antibodies (red) in vertical frozen retinal sections of developing retina (p14 and p30) and of 3-month old C57BL/6J mice after 0 and 2 days in culture. DAPI counterstaining (blue) reveals the chromatin in relation to the location of 53bp1 and lamin B_2_ immunoreactivity in the nuclei. **(A)** Entire retina and magnifications of ONL **(B,D)** and INL **(C,E)** at 2 weeks of age. **(F)** Entire retina and magnifications of ONL **(G,I)** and INL **(H,J)** at 4 weeks of age. **(K)** Entire retina and magnifications of ONL **(L,O)**, INL **(M,P)** and GCL **(N,Q)** of 3 months old C57BL/6J mice. **(R)** Entire retina of 3 months old C57BL/6J mice taken into culture for 2 days and magnifications of ONL **(S,V)**, INL **(T,W)** and GCL **(U,X)**. Individual 53bp1 and lamin B_2_ immunoreactive nuclei are zoomed in 2-fold and displayed as inserts in the respective micrographs of different retinal layers **(C** + **E, G** + **I, L** + **O, M** + **P, N** + **Q)**. For detailed description see Results. Localization of 53bp1 pan nuclear staining with regard to the heterochromatin in cone photoreceptors in C57BL/6J mouse retina at p30 is presented in Supplemental Figure 2. IS, inner segments; ONL, outer nuclear layer; OPL, outer plexiform layer; INL, inner nuclear layer; IPL, inner plexiform layer; GCL, ganglion cell layer. Scales in **A–Q** = 20 μm, scales in **R–X** = 10 μm.

Double immunostaining of lamin B_2_ and 53bp1 allowed us to localize 53bp1 immunoreactivity within the nuclei. Chromatin counterstaining by DAPI made the localization of 53bp1 clearly distinguishable from the brightly labeled heterochromatin of the chromocenters. This is particularly important for mouse rod photoreceptors, since their chromatin structure is inverted, i.e., condensed in the nuclear center (Figures 9D,I,O,V).

In all investigated mouse retinal tissues, the nuclear lamina was clearly labeled in all nuclei throughout the retina, yet with different immunofluorescent intensities (Figures 9A–X). In general, there was an increase in the labeling intensity starting from low levels in rods and cones, through medium intensity in the INL and highest labeling in the GCL (Figures 9A,B,F,K,R–U). Apart from the nucleoli, 53bp1 was dispersed throughout the entire nucleus and surrounded by lamin B_2_ positive nuclear lamina (Figures 9C,H,M,N). Heterochromatin in nuclei of the INL and GCL is counterstained with DAPI (Figures 9E,J,P,Q,W,X). In all retinal layers, 53bp1 immunoreactivity was clearly located close to the nuclear lamina (Figures 9G,H,L–N). Interestingly, lamin B_2_ immunoreactivity was brighter around most 53bp1 immunoreactive nuclei compared to 53bp1 immunonegative nuclei, especially obvious in cones (Figures 9G,I,L,O). Irrespective of the age of the mouse retina, the same quality of lamin B_2_ and 53bp1 immunofluorescence was observed in the different retinal layers. In cone nuclei, 53bp1 immunoreactivity filled the space between the chromocenters and the nuclear periphery (Supplemental Figure 2A), omitting the central chromocenters (Supplemental Figures 2B,D).

## Discussion

In this study, we analyzed the distribution of γH2AX and 53bp1 proteins in all neurons of young, mature and degenerating retinae. Furthermore, we showed that the two proteins only occasionally co-localize and that in the majority of cases, DNA damage sensing does not seem to result in 53bp1 recruitment to repair foci, irrespective of the viability state of the retina.

DNA damage sensing happens through the appearance of H2AX at the DSB site and its subsequent phosphorylation, yielding γH2AX (Thiriet and Hayes, 2005; Yuan et al., 2010). Antibodies to γH2AX allow the visualization of a “focus” at the DSB site (Rogakou et al., 1999). These foci serve as sites for accumulation of other proteins involved in DSB repair, leading to the suggestion that the foci have roles in signal amplification and the accumulation of DNA repair factors that, in turn, facilitate chromatin remodeling, cell cycle checkpoint functioning, sister chromatid-dependent recombinational repair and chromatin anchoring to prevent the dissociation of broken ends (Redon et al., 2011). We observed intranuclear γH2AX immunoreactive foci commonly in the inner and outer nuclear layers of developing retina and less frequently in the mature retina. Especially in developing C57BL/6J and rd10 mice, the occurrence of several γH2AX foci per nucleus seemed quite common in the ONL and INL. The fact that γH2AX positive foci are still present in the adult retina is consistent with reports that the level of reactive oxygen species (ROS), a major cause for DSBs in non-replicating cells, is particularly high in retinas of nocturnal animals (Jarrett and Boulton, 2012; Frohns et al., 2014). In adult mouse retina, we were able to induce DSBs by potassium bromate (KBrO_3_). This led to increased numbers of γH2AX immunoreactive foci per nucleus in some photoreceptors. Hence, we were able to show DNA damage detection by immunohistochemistry for γH2AX in all investigated retinal tissues, inclusive of developing, degenerating and mature retinas.

In addition to discrete foci, we also observed γH2AX immunoreactivity as a pan nuclear staining. This pattern was found predominantly in individual nuclei of the INL and GCL in both the developing and mature retina. This pan nuclear staining was more frequently seen in degenerating retina, including the retina of rd1 and rd10 mice and in retinal explant culture. During the first two post-natal weeks, neuronal apoptosis is a common incident in the mouse retina (Young, 1984). As well, organotypic retina culture simulates the pathological condition of retinal detachment and photoreceptor cell death becomes more prominent during culture (Ferrer-Martín et al., 2014; Müller et al., 2017). The phenomena of retinal neuronal apoptosis and the previous finding of pan nuclear γH2AX staining associated with preapoptotic single kinase activity (de Feraudy et al., 2010) supports the finding that the γH2AX pan nuclear staining seen in these tissues is indicative of preapoptotic retinal neurons.

The DNA repair and mediator protein 53bp1 (Chapman et al., 2012), is recruited to DSB after enhancement of H2A and H2AX ubiquitinylation by RNF168 (ring finger protein 168) (Brandsma and Gent, 2012). Together with RAP80 (receptor-associated protein 80) (Stewart et al., 2009) it is involved in deciding the fate of the proceeding repair pathway by binding factors which are part of the NHEJ pathway (Ward et al., 2003; Ginjala et al., 2011). The mammalian protein 53bp1 is activated in many cell types in response to genotoxic stress, including DSB formation (Anderson et al., 2001; Rappold et al., 2001; Manis et al., 2004; Lukas et al., 2011). Previously, various studies reported function of 53bp1 as a tumor suppressor gene in breast cancer (Kong et al., 2015). In breast precancerous lesions and cancer tissue 53bp1 immunohistochemical staining was mainly localized in the nuclei of cells (Li et al., 2012; Kong et al., 2015). Also in colorectal cancer tissue nucleus staining of 53bp1 was considered positive (Bi et al., 2015). We consider nucleus staining as pan nuclear staining.

In all retinal tissue investigated in this study, we found 53bp1 immunoreactivity prominently in the nuclei of the inner half of the INL and in most if not all nuclei of the GCL. Apart from the nucleoli, 53bp1 was dispersed throughout the nucleus resulting in a pan nuclear pattern. This distribution of 53bp1 is perfectly in line with the description of 53bp1 immunoreactivity in the INL and GCL of un-irradiated adult mouse retina (Frohns et al., 2014). Frohns and colleagues investigated the presence of 53bp1 inside the INL after irradiation in a cell-type specific manner using immunefluorescent co-localization of cell type specific proteins and 53bp1. Thereby, amacrine cells, horizontal cells and Müller cells were shown to display 53bp1 pan nuclear staining. In the present study, we confirmed that the most brightly stained 53bp1 nuclei belong to amacrine cells and horizontal cells based on the location within the INL. The two innermost rows of retinal neurons in the INL consist of amacrine cells (Haverkamp and Wässle, 2000), which are laterally connected with bipolar cells and ganglion cells in the IPL (Kolb et al., 2007). The remaining nuclei in the outer half of the INL showed moderate to light 53bp1 immunoreactivity and appear more than likely to be nuclei of various types of bipolar cells (Haverkamp and Wässle, 2000), which are vertically oriented in the retina and connect the two plexiform layers, transmitting the visual information toward the ganglion cells (Kolb et al., 2007). Here we confirmed the occurrence of 53bp1 in horizontal cells and subpopulations of amacrine cells by double immunostaining of 53bp1 and calbindin in degenerating and wildtype mouse retina at 4 weeks of age following a piggy-back immune protocol (Haverkamp et al., 2003). Somata of the bipolar oriented Müller glia cells reside in the middle of the INL, between the distal bipolar cell somata and the proximal amacrine cell somata (Haverkamp and Wässle, 2000).

Frohns et al. (2014) did not find 53bp1 immunoreactivity in bipolar cells or in cells of the ONL, which is in contrast to the results presented here. At all ages investigated, we found 53bp1 immunoreactivity in cones distributed between the nuclear lamina and the two large central chromocenters. In the mouse retina, only 3% of all photoreceptors are cones (Jeon et al., 1998) and can be identified by their location close to the outer limiting membrane and their conventional heterochromatin organization, i.e., more than one chromocenter per nucleus (Solovei et al., 2009). Additionally, in rod photoreceptors, we found faint 53bp1 immunofluorescence, visible as a thin ring very close to the nuclear envelope, the location of the euchromatin in the inverted nucleus of rods (Solovei et al., 2009), in which the central part of the nucleus is taken up by one large chromocenter, the heterochromatin.

The differences concerning the detection of 53bp1 in photoreceptors and bipolar cells in our study and that of Frohns et al. (2014) might have resulted from the different treatment of retinal tissue. In our study, we used frozen sections of lightly fixed retinae (30–45 min fixation in 4% paraformaldehyde). Frohns et al. (2014) applied much longer fixation time (16 h) and retinal tissue was embedded in paraffin, both of which can result in a diminished antigen presentation in the retinal tissue (Osborn and Brandfass, 2004; Eberhart et al., 2012).

In addition to the pan nuclear staining, the 53bp1 antibody revealed some immunoreactive foci which we believe correspond with DSB repair foci within the heterochromatin (Rappold et al., 2001; Manis et al., 2004; Lukas et al., 2011; Frohns et al., 2014). Since only very few double labeled foci appeared in the INL and ONL, we conclude that 53bp1 is not regularly recruited to a DSB after phosphorylation of γH2AX in the ONL and INL.

Overall, we can only partly confirm in our study the observation of Frohns et al. (2014) in the irradiated mouse retina. They showed that in the INL, numerous double labeled repair foci were present in amacrine cells and horizontal cells, which are those cells that showed bright pan nuclear 53bp1 staining before irradiation in their study. Interestingly, after irradiation, pan nuclear staining of 53bp1 was completely gone (Frohns et al., 2014). In contrast, in the present study many γH2AX positive foci were found in nuclei with pan nuclear 53bp1 staining in the INL and GCL of all investigated retinae. It remains enigmatic why within the INL different cell types show different staining behavior concerning 53bp1 but at the same time demonstrated the same repair capacity in the study by Frohns et al. The fact that presence or absence of 53bp1 does not affect the DNA repair efficiency after irradiation in the INL leads to the conclusion that either a different DNA repair mechanism is active in murine post-mitotic retinal tissue or that the repair of DNA damage induced by ionizing radiation is independent of the presence of 53bp1 (Ward et al., 2004; Bunting et al., 2010). Taken together, our results in the mouse retina concerning co-localization of 53bp1 and γH2AX clearly differ from irradiation experiments with respect to the occurrence of 53bp1 repair foci (Rappold et al., 2001; Frohns et al., 2014).

In our study, potassium bromate treatment of adult mouse retina did not increase the number of γH2AX and 53bp1 double labeled foci in either ONL or INL. This finding also supports an alternative in the post-mitotic mouse retina to the DSB repair via the conventional NHEJ pathway. This is a surprising and noteworthy finding, since ordinarily DSBs are repaired by one of two main pathways: either homologous recombination (HR) or NHEJ (Chapman et al., 2012; McKinnon, 2013).

In summary, we observed different intensities of the 53bp1 immunostaining in specific cell types in the different retinal layers in mouse retinae at all ages from wildtype and retinal degenerating mouse lines, as well as in organotypic retina culture. With little of variation, 53bp1 was characterized by pan nuclear staining in amacrine and horizontal cells, the laterally connecting neurons in the retina. Cones showed 53bp1 immunoreactivity distributed between the nuclear lamina and the two large central chromocenters, which we viewed as pan nuclear staining. In the ONL and INL of the developing retina and in retinal explant culture, γH2AX positive DSBs were found more numerously compared to adult retina. Most interestingly, we could show that the two proteins do not co-localize regularly in repair foci and that in the majority of cases, DNA damage sensing does not seem to result in 53bp1 recruitment, irrespective of the viability state of the retina.

In conclusion, our data indicate that DNA double strand breaks are sensed by phosphorylation of H2AX in all neurons of the retina, but this not necessarily leads to the recruitment of 53bp1 to repair foci, indicating the presence of alternative sensing and repair proteins. This observation warrants further investigation into the DNA repair pathway state in post-mitotic neurons of the retina and the central nervous system in general.

## Author contributions

BM and KS contributed conception and design of the study. BM acquisition, analysis, editing, and interpretation of data for the study. BM drafted the manuscript. KS and NE were revising it critically for important intellectual content. BL read the manuscript critically. BM Agrees to be accountable for all aspects of the work in ensuring that questions related to the accuracy or integrity of any part of the work are appropriately investigated and resolved.

### Conflict of interest statement

The authors declare that the research was conducted in the absence of any commercial or financial relationships that could be construed as a potential conflict of interest. The reviewer SB and handling Editor declared their shared affiliation.

## Abbreviations

DSBs: double strand breaks
53bp1: p53 binding protein 1
γH2AX: phosphorylated form of H2AX
PRs: photoreceptors.
