# Supplementary material for: Detection of DNA Double Strand Breaks by γH2AX Does Not Result in 53bp1 Recruitment in Mouse Retinal Tissues
Source: Front Neurosci. 2018 May 1;12:286. doi: 10.3389/fnins.2018.00286 (PMC5938408; doi:10.3389/fnins.2018.00286)
Supplement: Supplementary file 1 [file Image_1.pdf]

Supplementary Figures and Figure Legends:

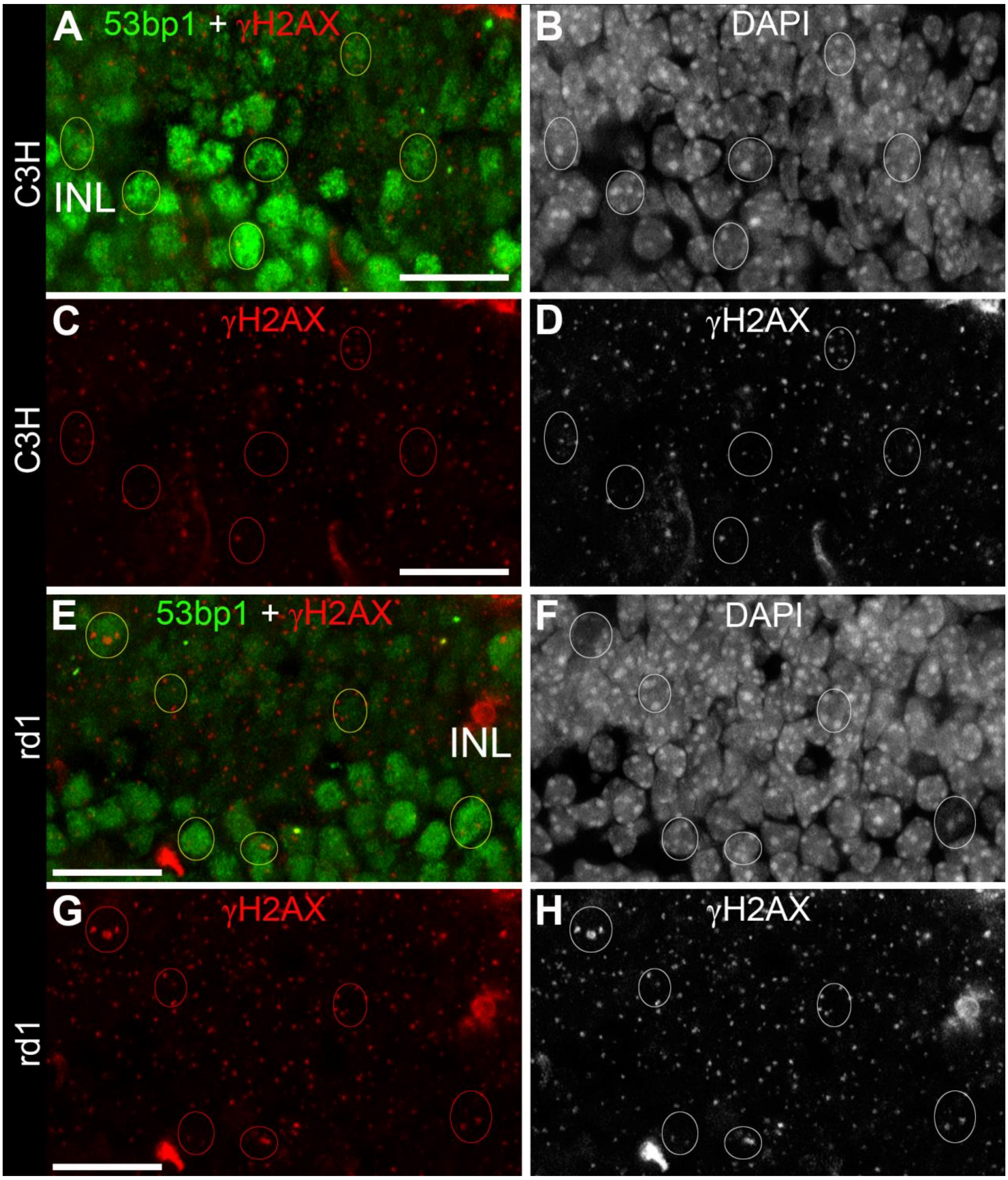

Supplemental Figure 1: Co-localization of γH2AX immunreactive foci and 53bp1 pan nuclear staining in the INL of C3H and rd1 mice at p13. (A, C) Double labeled sections of the INL displaying many 53bp1 immunoreactive nuclei with pan nuclear staining (some are marked by circles). Many γH2AX foci seem to be localized to the same nucleus as 53bp1. **(C, D, G, H)** Numerous γH2AX immunoporeactive foci are detectable in monochromatic micrographes, individual ones clearly assigning to 53bp1 pan nuclear immunoreactive nuclei. ONL: outer nuclear layer. All scales = 20 µm.

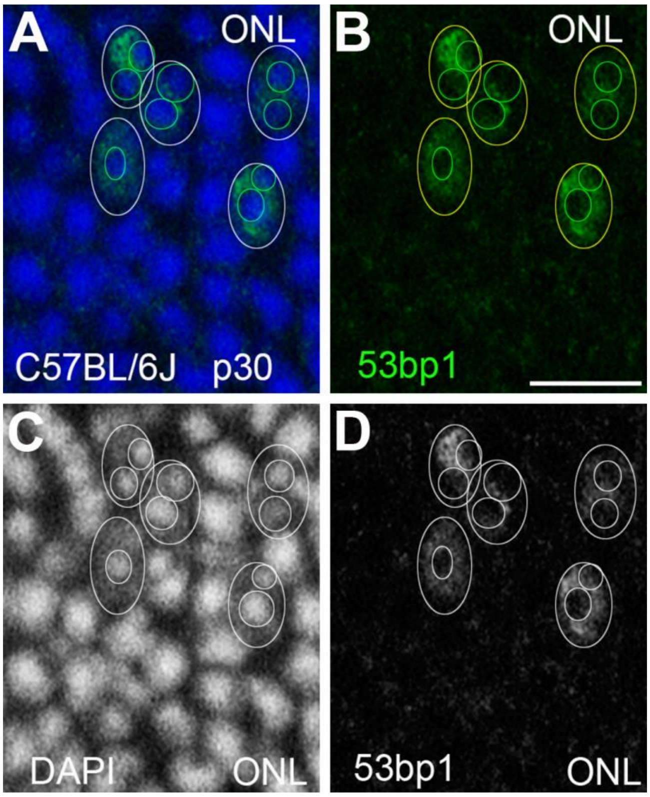

Supplemental Figure 2: **Localization of 53bp1 pan nuclear staining with regard to the heterochromatin in cone photoreceptors. (A)** 53bp1 immunoreactivity (green) is surrounding the chromocenters in cone photoreceptor nuclei. Heterochromatin is counterstained with DAPI (blue) in vertical frozen sections of the-one-month-old C57BL/6J mouse retina (p30). **(C, D)**: Grey scale micrographs correspond to color micrographs in **(A)** and **(B)** respectively. ONL: outer nuclear layer. Scales in B = 20 µm.

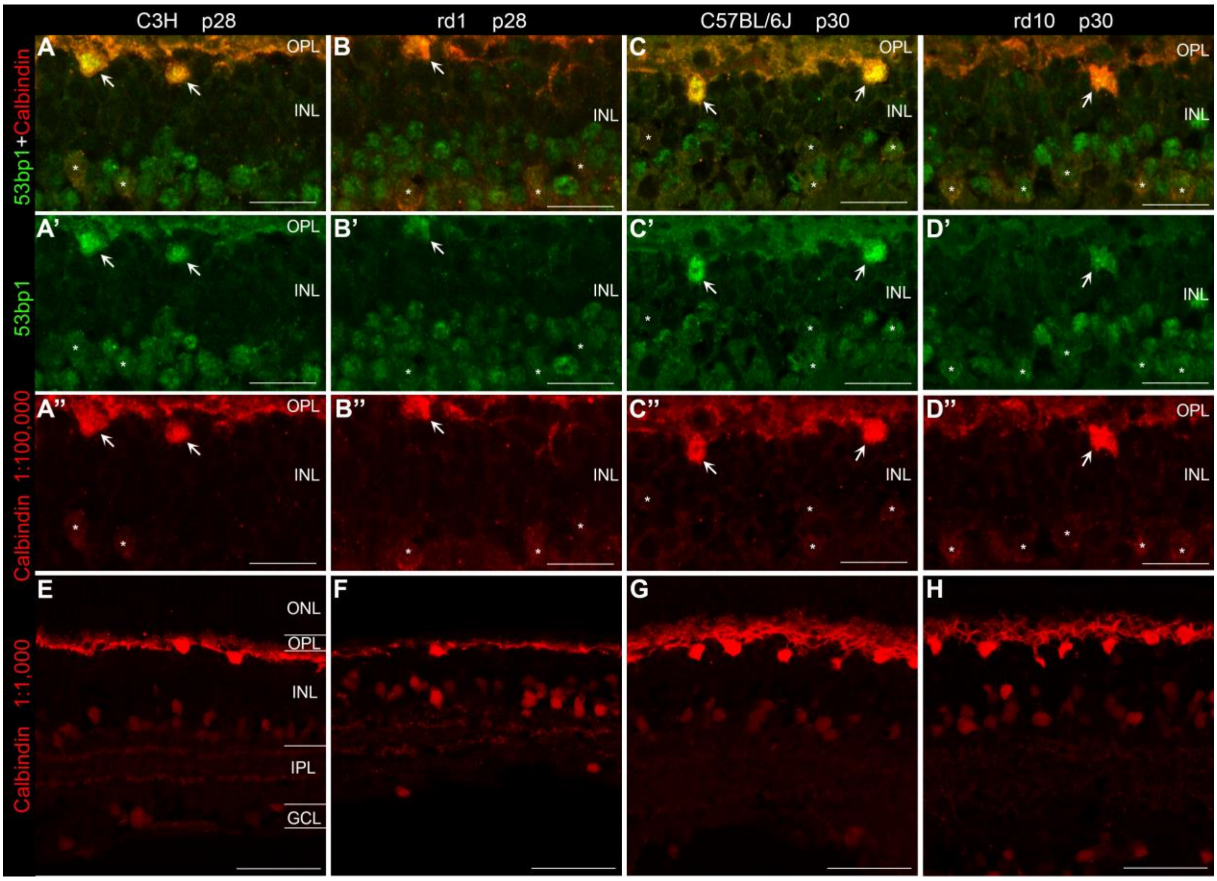

Supplemental Figure 3: Co-localization of 53bp1 and calbindin in degenerating and wildtype mouse retina at four weeks of age. Vertical frozen sections of retina of C3H, rd1, C57BL/6J and rd10 mice were double labeled with 53bp1 (green) and calbindin (red) antibodies. Only INL is shown at high magnification (A-D''). Due to the origin of both primary antibodies in the same host animal, a piggy-back immune protocol was followed (Haverkamp et al., 2003). In brief, sections were incubated first with one primary antibody (calbindin, 1:100,000), which was diluted hundred fold compared with the normal working dilution. The staining was then visualized by using a secondary antibody raised in goat and was intensified by following this with a tertiary donkey anti-goat antibody carrying the same fluorophore. The next step was incubation with the 53bp1 using the normal working dilution, followed by incubation in the secondary antibody. (E-H) Control immunostaining of calbindin antibody diluted 1:1,000 and applied in a single immunostaining. In all four mouse lines calbindin was localized in perikarya of horizontal cells in the distal INL and some amacrine cells in the proximal part of the INL. Horizontal cell perikarya (arrows) show strong immunolabeling for both primary antibodies visible as orange fluorescence (A-D). Some amacrine cells showing co-localization of 53bp1 and calbindin are marked by asterisks. ONL: outer nuclear layer, OPL: outer plexiform layer, INL: inner nuclear layer, IPL: inner plexiform layer, GCL: ganglion cell layer. Scales A-D'': 20 μm; scales E-H: 50 μm.
